# BRIDGING REGULATORY GAPS IN DIRECT-TO-CONSUMER HEALTH MONITORING TECHNOLOGIES FOR OLDER ADULTS

**DOI:** 10.1093/geroni/igae098.1112

**Published:** 2024-12-31

**Authors:** Anita Ho

**Affiliations:** University of British Columbia, Canada

## Abstract

This presentation explores unique ethical concerns that result from regulatory gaps in direct-to-consumer (DTC) AI health monitoring platforms, with a focus on the current landscape in the United States. Many older adults have multiple co-morbidities and are increasingly encouraged or even expected to continuously monitor their health, including by AI-powered predictive digital technologies. Some medically oriented AI-based algorithms can seek and receive regulatory approval as a medical device. Nonetheless, a much larger category of DTC AI health platforms includes wellness technologies that are not subjected to regulatory scrutiny or approval, even when many unregulated devices collect similar types of data as medical devices and vast amounts of health-related and personal information, blurring the line between medical and commercial data and products. Older adults are generally more risk averse and have limited digital, medical, and statistical literacy, and may also experience cognitive impairments. Enthusiasts often cite the potential of DTC health monitoring technologies to democratize health information and users’ well-being. However, the lack of governance for DTC applications raises unique and intersecting ethical concerns for the field of gerontology, particular around barriers for informed consent, lack of validation for product safety and clinical value, perpetuation of infantilization, non-transparent practices of data sharing, and potential impact of these technologies on familial care relationships and formal health care delivery. This presentation concludes with recommendations for regulatory considerations to uphold monitored older adults’ agency and well-being.

